# Multifaceted Properties of Usnic Acid in Disrupting Cancer Hallmarks

**DOI:** 10.3390/biomedicines12102199

**Published:** 2024-09-26

**Authors:** Mariola Gimła, Anna Herman-Antosiewicz

**Affiliations:** Department of Medical Biology and Genetics, Faculty of Biology, University of Gdańsk, 80-308 Gdańsk, Poland; mariola.gimla@phdstud.ug.edu.pl

**Keywords:** usnic acid (UA), antiproliferative activity, cancer treatment, cancer hallmarks, lichens

## Abstract

Cancer, a complex group of diseases marked by uncontrolled cell growth and invasive behavior, is characterized by distinct hallmarks acquired during tumor development. These hallmarks, first proposed by Douglas Hanahan and Robert Weinberg in 2000, provide a framework for understanding cancer’s complexity. Targeting them is a key strategy in cancer therapy. It includes inhibiting abnormal signaling, reactivating growth suppressors, preventing invasion and metastasis, inhibiting angiogenesis, limiting replicative immortality, modulating the immune system, inducing apoptosis, addressing genome instability and regulating cellular energetics. Usnic acid (UA) is a natural compound found in lichens that has been explored as a cytotoxic agent against cancer cells of different origins. Although the exact mechanisms remain incompletely understood, UA presents a promising compound for therapeutic intervention. Understanding its impact on cancer hallmarks provides valuable insights into the potential of UA in developing targeted and multifaceted cancer therapies. This article explores UA activity in the context of disrupting hallmarks in cancer cells of different origins based on recent articles that emphasize the molecular mechanisms of this activity.

## 1. Introduction

Cancer is a complex group of diseases characterized by uncontrolled cell growth and the ability of cells to invade surrounding tissues. The hallmarks of cancer are a set of fundamental characteristics acquired by cancer cells during the development of the disease. Initially proposed by Douglas Hanahan and Robert Weinberg in 2000, these hallmarks provide an organizing principle for understanding the complexity of cancer [1]. The original six hallmarks included sustaining proliferative signaling, evading growth suppressors, resisting cell death, enabling replicative immortality, inducing angiogenesis and activating invasion and metastasis [1]. Subsequently, two emerging hallmarks were added: reprogramming of energy metabolism and evading immune destruction. These hallmarks were underpinned by two enabling characteristics: genome instability and inflammation [2]. Although some reports attempted to emphasize other aspects of cancer biology, redefine or expand the concept of cancer hallmarks, it remained instrumental in rationalizing the diverse and complex nature of cancer, providing a framework for research and potential therapeutic interventions [3,4,5,6]. 

By targeting the hallmark of sustained proliferative signaling, therapies aim to inhibit the abnormal growth signals that drive cancer cells to divide uncontrollably [7]. Effective cancer therapy often involves strategies to disrupt the evasion of growth suppressors by cancer cells. Inhibiting invasion and metastasis is a key focus of cancer therapies aiming to prevent the spread of cancer cells to distant tissues and organs [8]. Therapeutic interventions targeting angiogenesis, a hallmark that involves the formation of new blood vessels, seek to deprive tumors of their blood supply and impede their growth [9]. Addressing the hallmark of replicative immortality, therapies aim to limit the ability of cancer cells to divide and escape the natural cellular aging process continuously [10]. Immune system modulation is a crucial aspect of cancer therapy, focusing on enhancing the body’s ability to recognize and eliminate cancer cells, countering the hallmark of immune evasion [11]. Therapies targeting resistance to cell death mechanisms are designed to induce apoptosis or other kinds of death in cancer cells. Combating the hallmark of genome instability and mutation involves therapies to enhance DNA damage repair and prevent the accumulation of genetic alterations and chromosomal instability that drive cancer progression [12]. Therapeutic approaches targeting the ability of cancer cells to sustain chronic inflammation aim to disrupt the tumor-promoting microenvironment and hinder cancer progression [13].

Natural products are invaluable sources of bioactive agents and the discovery of leads for drugs to treat different human diseases, including cancer. This is due to high biofunctionality and molecular diversity as well as superior efficacy and safety compared with synthetic compounds. As summarized by Newman and Cragg (2020), the total number of small molecules approved as antitumor drugs over the period 1946–2019 comes to 259, of which 79% are of natural origin [14]. They include natural compounds, their derivatives (usually semisynthetically modified) or compounds inspired by natural products (made by total synthesis, but the pharmacophore is from a natural product) [14,15]. Examples of clinically effective anticancer drugs of natural origin include paclitaxel from *Taxus brevifolia* or topotecan and irinotecan from *Camptoteca acuminate*, to name a few [14]. Advances in analytical techniques, such as non-targeted metabolomics based on mass spectral networking, DNA sequencing and high-throughput screening should speed up the identification of new active compounds in natural sources [16], while computational chemistry coupled with artificial intelligence and combinatorial synthetic methodologies enhance the development of anticancer drugs based on natural compounds [17].

Usnic acid (2,6-diacetyl-7,9-dihydroxy-8,9b-dimethyldibenzo[b,d]furan-1,3(2H,9bH)-dione, UA) is one of the most investigated bioactive compounds found in lichens. It occurs in nature as (–) and (+) isomers as well as a racemic mixture [18]. These enantiomers may exhibit different biological activities and interactions due to their distinct spatial arrangements, making them important considerations in pharmaceutical and biological applications [19,20]. UA has been studied for its various biological activities such as antibacterial, antiviral, antimycotic, antiprotozoal, anti-inflammatory, analgesic, neuroprotective and anti-cancer (reviewed in [19,21,22,23]). The anti-cancer activity of (–)-UA was reported in 1975 when Kupchan and Kopperman isolated this compound from *Cladonia* species and treated mice harboring Lewis lung carcinoma with (–)-UA at a dose range of 20–200 mg/kg and that extended the life of animals even to 52% over the untreated control group [24].

The purpose of this article is to summarize and upgrade the knowledge of UA activity toward cancer cells in vitro and in vivo. Thus, more recent publications are presented, especially ones investigating mechanisms of UA activity in the context of targeting cancer hallmarks. This work focuses on data reported on pure UA (either purified from lichen’s extracts or synthesized) and not on extracts containing this compound, its derivatives or formulations, although they are mentioned.

## 2. UA Inhibits Cell Proliferation and Induces Apoptosis of Cancer Cells

Cancer cells’ ability to proliferate and survive harsh conditions is connected with hallmarks such as sustained proliferative signaling, which is associated with the activity of oncogenes, evading cell cycle suppressive and proapoptotic signals and replicative immortality. UA has been shown to affect these features in cancer cells.

The main pathways signaling cells’ survival and proliferation encompass growth factors and their receptors, Hedgehog and Wnt signaling, to name a few. It was reported that (+)-UA reduced the transcriptional activity of β-catenin/LEF and c-jun/AP-1, the final effectors of Wnt and MAPK pathways, respectively. Thus, expression levels of c-myc, CD44 and cyclin D1, proteins crucial for cancer cell survival and proliferation, were reduced in A549, H1650, H1975 and H460 non-small-cell lung cancer cells treated with (+)-UA [25]. 

Numerous studies document that UA exerts antiproliferative potential inducing cell cycle arrest at either the G_0_/G_1_, S or G_2_/M phase and/or cell death through apoptosis or necrosis. 

For instance, Backorova et al. (2011) showed that UA induced cell cycle arrest in the S phase in A2780 (ovarian), HL-60 (leukemia) and HCT116 (colon cancer cells) and in some of these cells, G_2_/M phase arrest took place, which was dependent on dose (50 and 100 μM) and treatment time (48 or 72 h). Interestingly, the antiproliferative activity of UA did not depend on the presence of a tumor suppressor: p53 protein. This work also revealed that UA induced apoptosis, and its extent was cell-line specific [26]. Later, when investigating mechanisms of this activity, researchers found that UA decreased mitochondrial membrane potential (MMP) and increased reactive oxygen species (ROS) or reactive nitrogen species (RNS) levels, especially after 48 and 72 h of treatment (in up to 90–100% cells) and dose-dependent changes in p53, Bcl-2 and Bax levels, which correlated with apoptosis in A2780 ovarian and HT-29 colon cancer cells [27]. 

G_2_/M arrest may suggest that the microtubule dynamic is disturbed. However, the treatment of MCF-7 breast or H1299 lung cancer cells with UA (29 μM for 24 h) did not result in any morphological changes in microtubules or an increase in the mitotic index compared to the effects of vincristine or taxol, drugs targeting microtubules. These results suggested that the antineoplastic activity of UA is not related to alterations in the formation and/or stabilization of microtubules [28].

S phase arrest has been observed in human hepatoblastoma HepG2 treated with growing concentrations of UA (3.13, 6.25, 12.5, 25 or 50 μM). At the highest concentrations, the fractions of subG_0_/G_1_ cells were elevated, which were confirmed to be apoptotic cells. Lower viability was correlated with decreased levels of pro-survival proteins, such as Bcl-2 and Mcl-1 as well as Akt and p-Akt (Thr-308 and Ser-473), mTOR and p-mTOR (Ser-2448), p-S6K (Ser-371) and p-4E-BP1 (Thr-37/46). Additionally, UA elevated autophagy, which played a protective role as its inhibition by 3-methyladenine or chloroquine or downregulation of Atg7 potentiated apoptosis. Moreover, the autophagy-regulated activation of JNK played a protective role, and its inhibition increased apoptotic cell fraction [29].

Another study compared the response of HepG2 (HBV-negative) and SNU-449 (HBV-positive) hepatocellular carcinoma cell lines to UA. The lichen compound reduced viability in a dose- and time-dependent manner, and SNU-449 cells were more sensitive to UA than HepG2 cells. UA also induced cell cycle arrest at the G_0_/G_1_ (HepG2) or S and G2/M (SNU-449) phase and apoptotic cell death after 48 h treatment. Autophagy induction was detected after 36 h of treatment. The viability of HUVEC cells (normal endothelial cells) was not affected by UA tested in the concentration range of 6.25–100 μM, which shows the selectivity of UA toward malignant cells [30].

UA-induced decrease in pro-survival signaling pathways was also reported in other studies. Nguen et al. (2014) investigated the impact of *Flavocetraria cucullata* metabolites on a panel of noncancerous and cancer cell lines. UA at concentrations as low as 5 and 10 μM decreased p-Akt (Ser-473), p-ERK1/2 (Thr202/Tyr204 and Thr185/Tyr187) and p-c-Jun (Ser-63) in A549 lung cancer cells. UA at 10 μM modulated the epithelial–mesenchymal transition (EMT) markers: reduced mRNA for Snail, Twist and N-cadherin and elevated E-cadherin (at the transcript and protein level) in these cells, as well as reduced time-dependent migration, invasion and anchorage-independent growth of A549 and AGS gastric cancer cells. UA decreased the viability of prostate CWR22Rv-1, lung A549, colon HT29 and gastric AGS cancer cells and had no impact on the viability of four different cell lines representing normal cells. Depending on the concentration (25, 50 or 100 μM) and cell line, UA inhibited cell cycle progression after 24 h treatment in the G_0_/G_1_ or S phase (CWR22Rv-1 and A549) or in the G_2_/M phase (AGS and HT29) and induced apoptosis of cancer cells with an elevation of a Bax:Bcl-xL ratio, procaspase-3 and PARP cleavage, especially in AGS and CWR22Rv-1 (after 48 h of exposition) [31]. 

Ebrahim et al. (2017) showed that UA (15 and 25 μM) induced autophagy in breast cancer cells, which was accompanied by a decrease in mTOR activity reflected by a drop in p-Akt, p-4E-BP1, p-S6K in breast MCF-7 and MDA MB 231 cancer cells. UA also inhibited the motility and invasion of MDA MB 231 cells (at 10–30 μM and 10 μM, respectively) [32].

In gastric cancer cells, 100–400 μM UA induced G_0_/G_1_ arrest (in BGC823 cells) or G_2_/M (in SGC7901 cells) after 24 h treatment and apoptosis with the rise of the Bax:Bcl2 ratio (also in vivo) and caspase 3 activation as well as autophagy. Additionally, in vivo, UA (100 mg/kg i.p. for 11 days) was more effective than 5-fluorouracil (5-FU, 25 mg/kg) in the retardation of BGC823 tumor growth in mice [33].

In A-431 squamous carcinoma cells, UA also induced cell cycle arrest in G_0_/G_1_, apoptosis and necrosis (concentration tested within a range of 25–250 μM), which was connected with a reduction in MMP and reduced glutathione level, rise in ROS production, lipid oxidation and structural changes in DNA and surface lipids and proteins [34].

Mechanisms underlying G_0_/G_1_ arrest induced by UA have been investigated in A549 human lung carcinoma cells. The authors observed arrest at the G_0_/G_1_ phase in cells treated with 25, 50 or 100 μM UA for 24 or 48 h. It was accompanied by decreased levels of CDK4 and CDK6 cyclin-dependent kinases and cyclin D1 and increased levels of p21/Cip1, CDK inhibitor. UA treatment also enhanced cell death by up to twofold (24 h treatment) and eightfold (48 h treatment). While examining the cell death-associated molecular changes, authors observed that UA induced mitochondrial membrane depolarization and cleavage of PARP [35].

UA suppressed JAK1/2-Src-STAT3 and RAS-RAF-MEK-ERK pathways in HeLa cells. It led to a drop in the production of programmed death ligand 1 (PD-L1), which not only enhanced the cytotoxic activity of T lymphocytes toward cancer cells but also resulted in a decline in viability and clonogenic potential of HeLa cells, and it correlated with reduced levels of c-myc and cyclin D1. UA inhibited mTOR, leading to MiT/TFE nuclear translocation and enhanced lysosomal biogenesis [36].

Recently, using UA-linker-Affi-Gel, 14-3-3 proteins have been identified as UA targets. Interaction between these molecules led to the degradation of 14-3-3 by proteasomal and autophagy pathways in Caco2 colon cancer cells. As 14-3-3 proteins bind to numerous phospho-proteins, their elimination affects cell proliferation, invasion, metabolism and signaling pathways regulating cell survival. Authors showed that UA (10 μM) reduced levels of different proteins overrepresented in HCT-116 colon cancer cells expressing different isoforms of 14-3-3. Among the downregulated proteins were cyclin D1, cyclin B1 and p-Cdc2 (Tyr-15), which explains UA-induced G_0_/G_1_ arrest. Moreover, UA reduced levels of proteins phosphorylated at positions crucial for their activity such as p-mTOR (Ser-2488), p-Akt (Ser-473), p-STAT3 (Tyr-705), p-JNK (Thr183/Tyr185) and levels of EMT markers (Snail, Twist, N-cadherin and β-catenin). UA also reduced the activity of AP-1, STAT and NF-κB transcription factors, which were elevated by the overexpression of 14-3-3 isoforms in HEK293T cells [37]. 

Interesting mechanisms of UA activity have been noticed in human gastric and colon cancer cells treated with potassium usnate (KU), a water-soluble form of UA. The viability of a panel of cell lines (AGS, MNK45, SNU638, Caco2, HCT116 and HT29) was dose- and time-dependently reduced by KU. Moreover, 24 h treatment with KU at IC_50_ concentrations induced cell cycle arrest in the G_0_/G_1_ or S phase, depending on the cell line, which was accompanied by a drop in CDK4, cyclin D2 and the transient elevation of p21 protein levels. More detailed investigations based on gastric SNU638 and colon HCT116 cancer cells revealed that KU induced endoplasmic reticulum (ER) stress with the elevation of intracellular Ca^2+^, ROS and ER stress markers, such as BIP, PERK, IRE1α, p-EIF2a, CHOP and ATF3. ATF3 is a transcription factor controlling the expression of ER stress (such as ATF3 itself), cell cycle modulating (GADD45) and apoptotic genes (Bak, PUMA and DR5). Its activity appeared crucial for the KU cytotoxic effect. Downregulation of ATF3 by specific siRNA protected against the KU-induced elevation of Bak, p-BAD, PUMA, activation of caspase 3 and cell death. Moreover, KU (20 mg/kg i.p. injections for 16 days) applied to mice with CT26 metastatic colon cancer cells reduced the number of metastatic nodules in livers, elevating ATF3 and cancer cell apoptosis levels [38].

UA was shown to be a novel Pim-1 inhibitor (IC_50_ = 202 nM). This protein serine/threonine kinase is often overexpressed in hematopoietic malignancies and acts as an oncogene supporting myc-driven transcription, 4E-BP-1-dependent translation and inactivation of pro-apoptotic Bad. The study found that UA inhibited the proliferation of human HL-60 acute myeloid leukemia cells and K562 chronic myeloid leukemia cells (IC_50_ = 10 and 10.4 μM, respectively, after 3 days of treatment) and induced apoptosis. HL-60 cells were more responsive and after 18 h of treatment with 20 μM, the UA apoptotic fraction increased from 3% to over 30%, while such an amount of apoptotic K562 resulted from 48 h treatment with 60 μM UA. It was accompanied by the activation of caspase 3, 9 and 8, a decrease in anti-apoptotic Mcl-1, reduced p-eIF4E, p-4E-BP1 and p-Akt levels in both cell lines, as well as a decrease in c-myc, cyclin D1, p-Bad, Pim-1, MNK1 and increased p27 protein levels in K562 cells, which resulted from an inhibition of MNK1/eIF4F and Pim-1/4E-BP1 signaling pathways [39]. 

UA also exerted antiproliferative and pro-apoptotic effects in prostate cancer cells, hormone-independent DU-145 and PC-3 [40,41] and hormone-dependent LNCap cell lines [42]. In addition to features characteristic for apoptosis (Bax:Bcl-2 mRNA elevation, drop in MMP, caspase activation), a decrease in NFκB p50 at the protein level and *NFKB1* mRNA was reported in DU145 prostate cancer treated with 40 μM UA [41]. 

Another study investigated the mechanisms of the anticancer effects of (+)-UA from *Cladonia arbuscula* and (−)-UA from *Alectoria ochroleuca* on two human cell lines, T47D breast cancer cells and Capan-2 pancreatic cancer cells. The study found that both enantiomers were equally effective in inhibiting cell proliferation. (+)-UA at 10 µg/mL arrested cell cycle at G_0_/G_1_ after 24 h treatment and decreased MMP; however, apoptosis was not detected. Instead, necrosis was seen in Capan-2 cells treated for a longer (48 h) time [43].

The activity of UA was also investigated against OVCAR-3 and A2780 ovarian cancer cells. The study utilized real-time cell analysis and demonstrated the antiproliferative effect of UA for these cell lines with no impact on non-cancerous L929 cells. UA at a concentration of 20 μM inhibited the cell cycle at the G_0_/G_1_ phase and induced apoptosis of OVCAR-3 cells treated for 48 h. Evaluation of the expression of apoptosis-related genes showed that UA significantly upregulated *Casp-1*, *Casp*-*8*, *TRAF6*, *CHECK1*, *CHECK2*, *RIPK2*, *Bak1*, *Bag1*, *Bag4*, *BCL2A1*, *TNFRSF21*, *TP53*, *CIDEA*, *GADD45*, *BIRC3* and *5* and downregulated some genes of TNF and Bcl-2 family. It also blocked cell migration and invasion [44]. 

UA also modulated the expression of apoptosis-related genes of the apoptosis pathway in SKBR-3 breast cancer cells with significant elevation of mRNA for caspases 3, 4, 10, TRAF 5 and 6, numerous TNF family members, APAF1, Bik, Bak1, Bax, Bok, MCL1, p53, Chek1, Chek2, DAPK2, RIPK2, GADD45A and reduction in mRNA for Bcl2, Bcl2L11, Bag1, Bag4. Moreover, Bax, caspases 3 and 9 have also been significantly elevated at the protein levels in cells treated with 7.2 μM UA for 48 h. Notably, MCF-12A noncancerous breast epithelial cells, were resistant to UA used up to 10 μM concentration [45]. 

The impact of UA on breast cancer cells with attention to miRNA expression profile was investigated. MDA MB 231, MCF-7 and BT-474 cells were treated with UA (IC_50_ c.a. 13 μM) for 48 h and RNA was analyzed using microarrays. The authors identified differentially expressed miRNAs, and their number was cell-line-specific (67 in MDA MB 231, 8 in MCF-7 and 15 in BT-474). MiRNAs were almost unique to each cell line; however, their targets were discovered to play a role mainly in four pathways in all three cell lines: basal cell carcinoma, the neurotrophin signaling pathway, gap junction and the Hedgehog signaling pathway. Pathway enrichment analysis revealed that in MDA MB 231 cells, most targets of miRNA were transcripts involved in MAPK, Erb, PI3K-Akt and p53 pathways, while in BT-474, it involved Erb, mTOR signaling, focal adhesion and gap junctions. UA increased the level of has-miR-185-5p, miRNA downregulated in many cancers, which is connected with their chemoresistance [46]. This miRNA, when overexpressed in BT-474, induced G_0_/G_1_ cell cycle arrest and apoptosis (with no effect in MCF-12A noncancerous cells), which was connected with the upregulation of pro-apoptotic Bcl-2 members, caspases, kinases related to cell death, death receptors and the downregulation of antiapoptotic Bcl-2 [47].

Oncogenic long noncoding RNA urothelial cancer associated 1 (*UCA1*) was identified as another target of UA. UCA1 is regarded as an oncogene due to its stimulating effects on cancer cell proliferation, migration and invasion. It was shown to be upregulated in endometrial cancer tissue compared to normal tissue and contribute to cancer development. UA inhibited the dose- and time-dependently survival of Ishikawa endometrial cancer cells (IC_50_ = 51.76 μM after 48 h treatment), which correlated with a 3-fold decrease in UCA1 level [48]. 

In the search for the mechanisms of antiproliferative activity of UA against breast cancer cells, Zuo et al. (2015) found that it induced ROS generation in MCF-7 cells, which triggered the mitochondrial pathway of apoptosis with the activation of c-Jun-N-terminal kinase (JNK), an increase in the Bax:Bcl-2 ratio, a drop in MMP, the release of cytochrome c and caspase cascade activation. N-acetylcysteine (NAC) protected against these effects indicating that UA-induced ROS are responsible for them. UA given intraperitoneally inhibited tumor growth in a murine xenograft model with a dose of 100 mg/kg being more effective and less toxic to animals than cyclophosphamide (25 mg/kg bw) [49].

ROS induction was also observed in H520 and Calu-1 lung squamous cell carcinoma treated with (+)-UA (10, 20 or 40 μM). Authors found that it was caused by inhibition of mitochondrial respiratory chain complexes I and III and lower stability of nuclear factor erythroid 2-related factor 2 (Nrf2). It resulted in a drop in mRNA for heme oxygenase 1 (HO1) and NAD(P)H quinone dehydrogenase 1 (NQO1), enzymes engaged in protection against ROS. Mitochondria-targeted antioxidant Mito-TEMPOL partially protected against UA-induced oxidative stress, while the Nrf2 agonist tBHQ was more effective, also protecting against UA-induced apoptosis. In the xenograft model, UA at a dose of 50 mg/kg (i.p.) reduced tumor growth, which was blocked by NAC (in drinking water) and potentiated the anticancer activity of paclitaxel (10 mg/kg) [50]. 

Different preparations from lichens were tested against glioma cells. Acetone extracts from *Parmelia sulcata*, *Evernia prinastri* and *Cladonia unciali* more potently reduced the viability of A172 and T89G cells than pure compounds derived from these extracts, i.e., salazinic acid, evernic acid and UA; for instance, IC_50_ values for *C. uncialis* extract were approximately 11 and 3.9 µg/mL, respectively, while IC_50_ values for (−)-UA purified from this extract were 31.5 and 13 µg/mL, respectively. Although extracts revealed weak free radical scavenging and Cu^2+^ ions reducing activities in vitro, pure compounds had no antioxidant activities. All tested extracts and compounds, including (−)-UA, inhibited superoxide dismutase (SOD) activity and (−)-UA revealed inhibitory activity against glutathione reductase (GR) and glutathione peroxidase (GPx), enzymes engaged in ROS defense [51].

UA isolated from *Usnea cornuta* extract was concentration-dependently cytotoxic to MCF-7, A-549 and HeLa cells (IC_50_ values were 89, 84 and 48.7 μM, respectively, after 24 h of treatment). More detailed studies on HeLa cells revealed that UA caused a drop in MMP and GSH levels and increased ROS production and lipid peroxidation. It also induced autophagy and chloroquine, an inhibitor of late stages of autophagy, potentiated ROS production, depletion of GSH, lipid peroxidation and apoptosis induced by UA used at 25 or 50 μM concentrations [52].

Increased ROS production may lead to DNA damage. However, data on UA-induced genotoxicity in cancer cells are inconsistent. In a study conducted by Mayer et al. (2005), UA showed antiproliferative activity against MCF-7 breast cancer cells (estrogen receptor-positive, wild type for p53) and MDA MB 231 (estrogen receptor-negative, non-functional p53) with an IC_50_ of 18.9 and 22.3 μM, respectively [53]. The authors found that the antitumor activity of UA did not involve DNA damage or p53 activation. In MCF-7 cells treated with UA, although there was an accumulation of p53 and p21 proteins, the transcriptional activity of p53 remained unaffected. They also found that there was no phosphorylation of p53 at Ser-15 after treatment of MCF-7 cells with UA, suggesting that the oxidative stress and disruption of the normal metabolic processes of cells triggered by UA did not involve DNA damage. The property of UA as a non-genotoxic anti-cancer agent that works in a p53-independent manner was highlighted as a promising candidate for novel cancer therapy [53]. 

Emsen et al. (2018) found that UA, although much more toxic to U87MG glioblastoma cells than to primary rat cerebral cortex cells, PRCC (IC_50_ values by MTT after 24 h were 41.6 and 132.7 µg/mL, respectively), did not significantly elevate 8-hydroxy-2′-deoxyguanosine (marker of DNA oxidative damage) levels in cells treated with 2.5–40 µg/mL UA [54].

On the other hand, studies published in 2020 reported that UA induced DNA damage. UA (10–25 μM) applied to SNU-1 and AGS gastric cancer cells induced apoptosis, which was connected with increased Bax:Bcl-2, depolarization of the mitochondrial membrane and ROS elevation. ROS in UA-treated AGS cells were responsible for DNA double-strand breaks revealed by alkaline comet assay and an increase in γ-H2A.X, DNA-PKcs, p-ATM (Ser-1981), Chk2 and p53—markers of DNA damage response. Moreover, NAC protected against DNA damage and cell death [55]. 

Another study showed that UA induced DNA damage response involving ATM kinase and G_2_/M cell cycle arrest in RKO colorectal cancer cells pretreated with 400 μM H_2_O_2_. Phosphorylation of histone H2A.X, which is the marker of DNA double-strand breaks, or ATM activation, was elevated in cells treated with H_2_O_2_ and UA (0.5, 1, 5, 10 μM) compared with H_2_O_2_ alone; however, the authors did not show how UA alone impacts DNA integrity and DNA damage response. Interestingly, low concentrations of UA (0.5 and 1 μM) reduced ROS levels in H_2_O_2_-treated cells, while 5 and 10 μM UA—increased ROS levels [56]. 

Data obtained in vivo indicate that both (+) and (−)-UA enantiomers at doses of 100 or 50 mg/kg induced DNA damage observed as increased tails in comet assays in the liver and kidneys of mice. Interestingly it was observed only 1 h after oral administration and not detected after longer times, probably due to rapid DNA repair. The authors also observed increased lipid peroxidation in cells, thus concluding that oxidative stress induced by UA might be involved in the genotoxicity of this compound [57].

DNA damage induced by UA and detected by comet assay was also reported in KB oral carcinoma cells. It was accompanied by increased ROS, reduced MMP, antioxidant enzymes and GSH levels and induction of apoptosis [58].

In addition to DNA damage due to oxidative stress, UA might be a potential inhibitor of key enzymes involved in DNA synthesis and repair. UA has been reported to be a rather weak inhibitor of PARP1 and polymerase β activity (residual activity of 73–77% after incubation with 0.5 mM UA), and some of its derivatives appeared to be much more active [59]. 

## 3. UA Inhibits Angiogenesis, Cancer Cell Motility and Invasion

Growth of tumors depends on angiogenesis; thus, the inhibition of this process can be an effective anticancer strategy. It has been demonstrated that UA inhibited angiogenesis in vivo based on a chick embryo chorioallantoic membrane assay, in a VEGF-induced mouse corneal angiogenesis model and in mice xenografted with Bcap-37 breast cancer cells [60]. Based on in vitro research, it has been shown that UA dose-dependently (1, 10, 20, 50 μM) inhibited activating phosphorylation of VEGFR2 and VEGFR2-mediated MEK/ERK1/2 and Akt/p70S6K signaling pathways in HUVEC endothelial cells, which resulted in a drop in cell proliferation, migration and tube formation and induction of apoptosis [60]. 

(−)-UA also inhibited HUVEC cell viability (IC_50_ after 48 h was 50 μM) and tube formation at concentrations 50 μM and higher (100 and 200 μM) [61]. It has also been shown that UA reduced VEGF and MMP-9 levels, which was partially dependent on the reduction in PD-L1 in HUVEC cells. These effects resulted in decreased tube formation, migration and the invasive potential of endothelial cells [36].

An article published in 2016 reported that (+)-UA inhibited A549, H1650 and H1975 non-small cell lung cancer cell migration and invasion at a concentration of 5 μM. The authors showed that (+)-UA decreased the level of active, GTP-bound Rac1 (by 22% compared with control) and GTP-RhoA (by 40% compared with control), which are crucial for cell motility regulation. Moreover, UA potentiated the activity of cetuximab, monoclonal antibodies against EGFR used for metastatic colon and lung cancer patients, in reducing the invasive potential of A549 cells [25]. The same team reported the antiproliferative activity of UA using a panel of colorectal cancer cell lines. At the concentration of 5 μM, UA inhibited invasion of Caco2, HCT116 and CT289 cells in vitro; however, it had no effect in the murine orthotopic liver metastasis model (applied at 5 or 10 mg/kg, 6 or 10 times within two weeks, i.p.), while its water-soluble potassium salt (UK) was more effective in vitro and in vivo. Both UA and UK decreased the mRNA levels of EMT markers, such as Twist, Snail, Slug, Zeb2 and N-cadherin in Caco2 cells [62].

SCF induces migration of c-KIT-containing colorectal cancer cells. It has been shown that (+)-UA at a concentration lower than 10 μM inhibited the SCF-induced migration of HCT116 and LS174 cells. The mechanism underlying this activity relied on the downregulation of c-Kit gene transcription mediated by sumoylation of Transcription Factor AP-2 alpha (TFAP2A) by upregulated UBC9 and degradation of c-KIT protein due to the induction of autophagy. This, in turn, resulted from decreased ATP level and inhibition of mTOR by 8 μM (+)-UA in HCT-116 cells. Caspases 3 and 7 were not activated by (+)-UA; however, the cell’s membrane was permeabilized, and LDH release after 48 h treatment increased, which suggests necrotic cell death [63].

UA inhibited the motility of prostate (DU145) and melanoma (HTB-140) cells, and it was connected with dose-dependent (at 10 or 25 µg/mL) rearrangements of the actin cytoskeleton [40].

It has been shown that the overexpression of PD-L1 in HUVEC endothelial cells led to the elevation of pro-angiogenic proteins, VEGF and MMP-9, enhanced tube formation, migration and invasion; however, UA (100 μM) protected against these processes decreasing PD-L1 level [36].

## 4. UA Facilitates the Immune Destruction of Cancer Cells

The programmed cell death 1 (PD-1) and its ligand PD-L1 are responsible for apoptosis and the exhaustion of T cells. PD-L1 is often elevated in cancer cells, which leads to avoidance of their destruction by immune cells. UA at 10, 30 and 100 μM concentrations has been shown to decrease PD-L1 levels in HeLa cervical cancer, A549 lung cancer, HCT116 colorectal cancer and liver cancer Hep3B cells, even if TNF stimulated production of PD-L1-α. It correlated with the enhanced cytotoxicity of co-cultured T lymphocytes toward HeLa, SiHa and CaSKi cervical cancer cells and higher production of TNF-α and ITF-γ by T cells. Authors identified mechanisms underlying diminished production of PD-L1 as reduced STAT3 and Ras signaling pathways, suppression of mTOR and subsequently increased MiT/TFE transcription factor translocation to the nucleus, enhanced biogenesis of lysosomes and proteolysis of PD-L1 [36]. 

It has been shown that lichen-derived extracts and compounds, including UA, possess inhibitory properties related to kynurenine pathway enzymes. (−)-UA at 100 µg/mL reduced by almost 22% indoleamine 2,3-dioxygenases 1 (IDO1), an enzyme involved in the conversion of L-tryptophan to L-kynurenine [51]. Metabolites of this pathway are crucial for the suppression of anti-tumor immune responses and IDO1 is highly expressed in multiple types of cancer [64,65]. 

## 5. UA Acts against Tumor-Promoting Inflammation

The anti-inflammatory activity of UA has been recently nicely summarized in publications by Wang et al. [23] and Pazdziora et al. [66]. However, it is worth mentioning a few studies in the context of cancer.

It has been shown that lichen-derived extracts and compounds, including UA, possess inhibitory properties related to kynurenine pathway enzymes. (−)-UA at 100 µg/mL reduced indoleamine 2,3-dioxygenases 1 (IDO1). It also strongly inhibited COX-2 (up to almost 60%), indicating its anti-inflammatory activity. Moreover, it inhibited hyaluronidase with IC_50_ = 500 µg/mL being more potent than β-escin used as a standard in this assay [51]. Hyaluronidase is responsible for generating low molecular weight hyaluronan, which displays pro-inflammatory properties, such as stimulation of macrophage activation and production of cytokines [67]. Another study by this team compared activity of (+)-UA and (−)-UA enantiomers showing that right-handed enantiomer is a slightly more potent (IC_50_ = 644.5 and 676.3 mg/mL, respectively) inhibitor of hyaluronidase [20]. This work also presented that both enantiomers (although to a different extent) decreased levels of pro-inflammatory molecules, such as Toll-like receptor 4 (TLR4), cytosolic phospholipase A2 (cPLA2), cyclooxygenases COX-1 and COX-2 in LPS-stimulated RAW 264.7 macrophages, as well as the release of nitric oxide (NO) and weakly TNF-α and IL-6 [20].

Another study investigated the anti-inflammatory effects of UA in MCF-7 breast cancer and found that it plays a crucial role in regulating the inflammatory response. UA dose-dependently decreased levels of NO, prostaglandin PGE2, cytokines IL-2, IL-6, CXCL10, CXCL8, CCL2, MCP-1 and TNF-α, as well as growth factor VEGF. Moreover, it downregulated the expression of genes coding for cyclooxygenase2 (COX-2) and inducible nitric oxide synthase (iNOS). At the same time, UA revealed pro-oxidant activity in cancer cells as it reduced glutathione and increased malondialdehyde (MDA) levels [68].

The reduction in pro-inflammatory proteins such as TNF-α, NF-κB or IL-6 was also reported in KB oral carcinoma cells treated with UA at concentrations of 10, 20 or 30 μM [58]. Moreover, the same team showed chemopreventive properties of UA as it protected against DMBA-induced oral squamous cell carcinoma in hamsters. Mechanisms underlying this activity were the suppression of inflammatory (COX-2 and iNOS) and proliferation markers (cyclin D1 and PCNA) induced by carcinogen as well as the upregulation of antioxidant levels or activity and the modulation of liver detoxification enzyme levels [69].

## 6. UA Deregulates Energetics in Cancer Cells

As some reports indicated that UA at high concentrations might be hepatotoxic, extensive research was performed to identify mechanisms underlying this activity. Data obtained on rodent primary hepatocytes or isolated mitochondria revealed that UA toxicity might be related to disturbed metabolism, particularly mitochondria functioning and drop in ATP level. These effects concerning normal non-cancerous cells have been beautifully presented in recent review articles [70,71]; however, it is worth mentioning a few studies. 

UA is a lipophilic weak acid; thus, it can easily pass the mitochondrial membranes, and in the matrix, it releases a proton resulting in the generation of a usniate anion. It diffuses into the intermembrane space, binds a proton and UA is restored. This cycling might cause a proton leak that could dissipate the proton gradient across the membrane, changing the mitochondrial membrane potential. The protonophoric activities of UA were documented using artificial planar bilayer lipid membranes and isolated rat mitochondria [72]. The analysis of biochemical profiles of rat primary hepatocytes showed that high doses of (+)-UA (10 or 30 μM) decreased ATP levels. It was connected with the depletion of glycogen stores, a drop in glycolysis and the tricarboxylic acid (TCA) cycle. Moreover, the mechanism of UA action resembled the action of mitochondrial uncoupler, FCCP, which supported the idea that UA is a proton carrier [73]. It has also been demonstrated that UA can carry calcium ions across liposomal, mitochondrial and erythrocyte membranes, thus behaving like a calcium ionophore [74].

UA has also been shown to affect the mitochondrial function in cancer cells. Numerous studies (described in the previous paragraph) show that UA causes a drop in MMP in cancer cells, which is connected with apoptosis. However, in some models, although UA decreased the proton gradient across the mitochondrial inner membrane, no release of cytochrome c was observed. UA as a lipophilic weak acid is supposed to act as a proton shuttle and directly dissipate mitochondrial inner membrane potential, affecting oxidative phosphorylation. Indeed, it was reported, that UA (5 and 10 µg/mL) decreased ATP level in T47D breast cancer cells after 24-h exposition. It activated AMPK, decreased mTOR/S6K signaling, upregulated p-eIF2α and induced autophagy. However, autophagy flux was impaired due to the disruption of lysosomal acidification and thus degradative processes did not occur [75]. Moreover, it was shown that UA inhibited the mitochondrial respiratory chain complexes (I and III) in lung squamous carcinoma cells, leading to a decrease in ATP production and an increase in the production of ROS [50]. Interestingly, the same team showed that UA affected lysosomal function in breast cancer cells.

Inhibition of glycolysis and mitochondrial respiration was also observed in CaCo2 and HCT116 colon cancer cells. UA at 2.5, 5 and 10 μM concentrations inhibited glycolysis (basal and compensatory) and mitochondrial functioning (basal respiration, ATP production, proton leak), which correlated with decreased expression of metabolic genes (*SLC2A1*, *SLC2A4*, *HK2*, *PFK1*, *GPI*, *ALDOA*, *PGK1*, *ENO1*, *PKM2*, *LDHA*, *CDH4*, *SRC1*, *PGC-1a*, *TFAM*, *PKM1*, *PDK1*, *ASCT2*, *SLC7A5*, *SLC7A7*, *GLS1*) and drop in SLC2A1, HK2, PKM2 and LDHA proteins, even when these processes were elevated by the surplus of an isoform of 14-3-3 [37].

**Figure 1:**
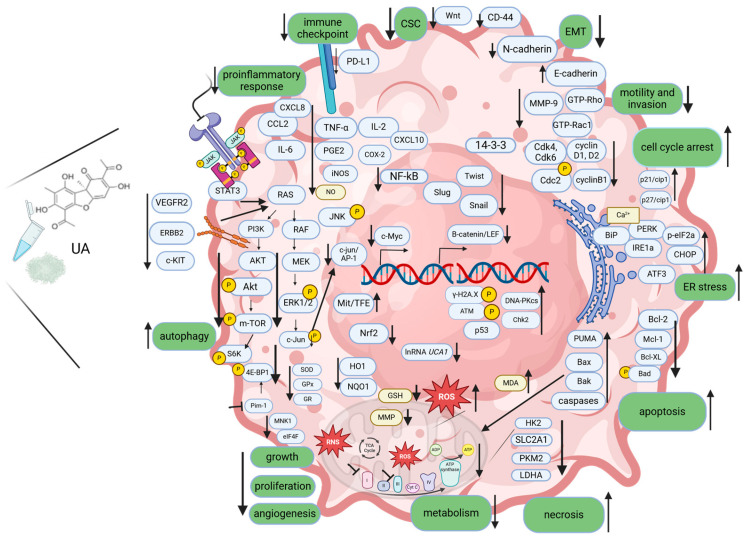
The mechanisms of action of UA in disrupting cancer hallmarks. UA inhibits cell proliferation, induces apoptosis of cancer cells, inhibits angiogenesis, cell motility and invasion, facilitates the immune destruction of cancer cells, acts against tumor-promoting inflammation and deregulates cellular energetics. Additionally, UA induces ER stress and autophagy and inhibits EMT and stem cell features. Created with BioRender.com.

Results of research investigating mechanisms of UA activity toward cancer cells in vitro and in vivo are summarized in Figure 1 and Table 1. 

It is worth mentioning that UA’s activity was documented against cancer cells of different origins, considering both differentiation stage (epithelial, such as adenocarcinomas and squamous cell carcinomas, and nonepithelial, such as leukemia) and embryonic origin (derived from ectoderm, for instance, breast cancer; neuroectoderm such as melanoma; endoderm, for instance, liver, colon or prostate or mesoderm such as endometrial or hematopoietic cancers). However, anti-tumor potential in vivo was validated only in the case of lung, breast, gastric and colorectal tumors (Table 1). 

## 7. Combination Therapies Using UA 

Increasing knowledge on mechanisms underlying the anticancer activity of UA is inclined toward combination therapies using UA and clinically approved drugs. Such combinations aim to enhance the anticancer effect and reduce the toxicity of a drug. 

The synergistic effect was observed when UA (at 12.5 or 50 μM concentration) was combined with low concentrations of tamoxifen, the selective estrogen receptor modulator), or enzalutamide (a second-generation androgen receptor inhibitor) for treating hormone-dependent breast MCF-7 or prostate LNCaP cancer cells, respectively. UA potentiated cell cycle perturbations and apoptosis induced by drugs. These effects were also observed in non-cancerous cells, however, at different extents than in cancer cells. Mechanisms of synergistic activity were not explored [77]. The same team reported that the combination of UA with sorafenib, a drug used in systemic chemotherapy of hepatocellular carcinoma, acted synergistically toward HepG2 and SNU-449 cells. Sorafenib at lower concentrations was not toxic to normal cells but when combined with UA more effectively arrested the cell cycle and induced apoptosis than any compound used alone and at the same time was less toxic to the HUVEC cell line [78].

It has also been shown that 5 μM UA and an anti-EGFR antibody, cetuximab, more effectively inhibited the invasive potential of A549 lung cancer cells than any of the compounds alone [25]. UA at a concentration of 15 μM enhanced the activity of paclitaxel at 0.1 μM toward H520 and Calu-1 lung cancer cells in vitro. Moreover, UA at a dose of 50 mg/kg (i.p.) reduced H520 xenograft tumor growth in vivo and potentiated the anticancer activity of paclitaxel (10 mg/kg) [50].

Another in vivo study investigated the efficacy of (+)-UA and bleomycin combination on hepatoma H22-bearing mice. Bleomycin is widely used to treat malignant ascites; however, it causes pulmonary fibrosis, which is connected with excessive inflammatory response and oxidative stress in lung tissue. UA (25, 50, 100 mg/kg, p.o.) combined with bleomycin (15 mg/kg, i.p.) revealed significantly better effectiveness than bleomycin alone in reducing ascites fluid, inhibiting ascites cell viability, arresting the cell cycle at G_0_/G_1_ phase and promoting apoptosis. It was associated with the transcriptional upregulation of p53/p21 and downregulation of cyclins E1 and D1. Moreover, UA reduced the side effects of bleomycin, including lung tissue damage. It was connected with a reduction in MDA, hydroxyproline (HYP), TNF-α, IL-1β, IL-6, TGF-β1 and p–Smad2/3 and an increase in SOD and Smad7 levels in lung tissues of H22-bearing mice treated with bleomycin [79]. 

More recently, the combination of (+)-UA or (−)-UA with doxorubicin was tested on HTB140, A375 and WM793 melanoma cells. The synergistic, additive or antagonistic effects were observed depending on the cell line, doses used and treatment time. Interestingly, UA (especially (−)-UA) sensitized to the drug WM793 cells, which are quite resistant to doxorubicin [20]. 

## 8. Bioavailability and Pharmacokinetics

The absorption, distribution, metabolism and excretion (ADME) are important factors in understanding the pharmacokinetics and potential toxicity of the new drug, and in the case of UA such data are limited. The available literature provides some insights into the dosage and administration of UA, particularly in the context of its pharmacokinetics and toxicity. For instance, studies conducted on rabbits indicate that plasma D(+)-UA levels following intravenous (5 mg/kg) administration showed a triexponential elimination with a mean terminal half-life of 10.7 ± 4.6 h. Following oral administration at a dose of 20 mg/kg the UA peak plasma concentration of 32.5 ± 6.8 g/mL was reached after 12.2 ± 3.8 h, and a mean terminal half-life was 18.9 ± 2.9 h. The mean absolute bioavailability of UA administrated orally was 77.8% [80].

Studies in rats administered intraperitoneally with 25 mg/kg of D(+)-UA have demonstrated that the molecule is distributed in different tissues, with a higher concentration found in lungs and liver, followed by blood (an average tissue-to-plasma concentration ratio was 1.777, 1.503 and 1.192, respectively). Moreover, approximately 99.2% of UA was bound to plasma proteins and showed albumin concentration-dependent binding (up to 6.5 g/L of albumin) [81], which was similar to results obtained by others showing that more than 99% of UA is bound in human or rat plasma [82].

UA metabolism has been studied in vitro using human plasma, hepatocytes and liver subcellular fractions. Three monohydroxylated metabolites and two glucuronide conjugates of UA were identified after incubation with human liver S9 fraction using liquid chromatography/mass spectrometry (LC/MS). Hepatic clearance of UA was estimated as 13.9 mL/min/kg and it was shown that UA is primarily metabolized by CYP1A2 while conjugation of UA with glycuronic acid depends on UGT1A1 and UGT1A3. Moreover, the study revealed that UA is a potent inhibitor of CYP2C19 and CYP2C9 and a less potent inhibitor of CYP2C8 and CYP2C19 [82].

The trapping assay with glutathione and UPLC-MS/MS analysis was used to elucidate reactive metabolites of UA in human, rat and mice liver microsomes. Authors found dehydrogenated and hydroxylated UA metabolic adducts with glutathione. These reactive adducts and/or depletion of GSH by UA might be related to UA hepatotoxicity. Interestingly, differences in metabolites were identified between human and rodent models and between (+) and (−)-UA enantiomers in human microsomes [83].

A more recent study investigated the metabolism of UA and its relationship to toxicity based on in vitro experiments using human liver microsomes, rat liver microsomes and S9 fraction combined with UPLC-Q-TOF-MS for metabolite identification. The authors identified 14 phase I metabolites and 4 phase II metabolites of UA and found that the key UA metabolizing enzymes are CYP2C9, CYP3A4, CYP2C8 and UGT1A1. UA was not toxic to human primary hepatocytes when applied at 0.01–25 μM concentrations for 48 h; however, it was toxic to mouse 3T3 fibroblasts (IC_50_ = 7.4 μM). Using a model of coincubation of human liver microsomes with 3T3 cells, authors found that UA (1–50 μM) or its metabolites were not toxic to 3T3 cells, at least after a short 4 h exposition; thus, they concluded that UA cytotoxicity might be related to chronic exposure [84].

Data on in vivo toxicology are scarce. Acute toxicity has been only determined for mice and rabbits, and 50% lethal dose (LD_50_) values in the case of oral applications were 838 mg/kg and > 500 mg/kg, respectively [85]. Intraperitoneal injections of UA suspension at a dose of 15 mg/kg/day for 15 days in male Swiss mice caused a hepatic dysfunction as revealed by a high level of serum transaminase and histological observation of necrotic areas in the livers. 

Data in humans are limited to reports on cases of severe hepatotoxicity (hepatonecrosis, fulminant hepatic failure and other complications) after taking dietary supplements containing UA, such as Lipokinetix, a weight loss formula [86].

## 9. Perspectives for the Use of UA as an Anticancer Drug

As shown in previous paragraphs, UA is effective in targeting cancer hallmarks; however, data from in vitro and in vivo models indicate that rather high concentrations of UA have to be applied, which might be toxic to healthy cells, especially hepatocytes. Another problem with the use of UA is its low water solubility. Thus, extensive research efforts are aimed in at least two directions: to obtain knowledge on structure–activity correlations and receive UA derivatives with enhanced activity and better selectivity toward cancer cells and to improve UA bioavailability by enhancing its solubility in water or delivery into the cells [87].

Numerous modifications to the UA structure have been reported and screened for antiproliferative activity (rev. in [87]). Some UA derivatives reveal much better activity than the parent compound, and molecular mechanisms of their action, as well as toxicological and pharmacokinetic studies, are important for their further clinical development. For instance, our results with a pyrazole derivative of UA, referred to as compound 5, have demonstrated superior anticancer activity compared to the parent compound. This derivative has shown significant inhibitory effects on the growth and proliferation of pancreatic cancer cells both in vitro (IC_50_ = 0.90–1.35 µg/mL after 48 h treatment) and in animal models. The compound induced the release of calcium ions from the ER, leading to ER stress in cancer cells. It also causes G_0_/G_1_ cell cycle arrest and cell death. When tested in nude mice with xenografted pancreatic cancer cells, the UA derivative 5 successfully inhibited tumor growth without causing apparent toxicity to the kidneys or liver [88].

The hydrophilic potassium salt of UA (KU) shows more favorable characteristics than the parent compound, which was comprehensively presented in the review article by de Araujo et al. [89]. Results by Yang et al. clearly indicate that the bioavailability of KU, measured as the amounts in tumor, liver and plasma of CT26 syngeneic tumor xenograft-bearing mice after oral administration (30 mg/kg), was higher than that of UA. Moreover, KU was more potent than UA in the inhibition of invasiveness of the majority of colorectal cell lines in vitro, and at a dose of 20 mg/kg (i.p.), it significantly decreased liver metastasis in an orthotopic murine colorectal cancer model [62]. In the KU acute oral toxicity tests in Swiss Webster mice, LD_50_ was evaluated as >200 mg/kg indicating much lower toxicity than UA [90].

Another way to improve the therapeutic index of UA is to develop drug delivery systems. Data on UA encapsulated into lipid-based nanocarriers (liposomes, nanoemulsions), polymeric nanocarriers or microparticles (of different structures and compositions) and nonorganic nanoparticles (magnetic or diamond) are comprehensively presented and discussed in a recent review by Zugic et al. [91]. For instance, Farzan et. al. developed a novel UA delivery system, where UA was encapsulated within nanoparticles of biodegradable gliadin (GNP) functionalized with hyaluronic acid (HA), which targets CD44 receptors overexpressed on breast cancer cells. This approach allowed for the targeted delivery of UA specifically to breast cancer cells, increasing efficacy and reducing side effects (UA IC_50_ = 120.04 μM, while UA-loaded nanoparticles HA-UA-GNPs IC_50_ = 0.56 μM and UA-GNPs IC_50_ = 92.64 μM). Tumor growth in mice treated with HA-UA-GNPs was significantly reduced compared with tumors in UA-GNPs or free UA-treated animals. The study demonstrated successful in vitro and translational research, bridging laboratory findings with potential clinical applications [76]. 

## 10. Overall Conclusions and Future Directions

Presented data indicate the versatility of UA in targeting different cancer hallmarks, including the inhibition of abnormal growth signals that drive uncontrolled cell division; induction of apoptosis or other cell death programs; disruption of cancer cell metabolism; inhibition of the new blood vessels formation which are crucial for the sustained growth of tumors; inhibition of migratory and invasive potential of cancer cells; the immunomodulatory potential in mobilizing body’s immune response against malignant cells; and anti-inflammatory properties, which can be beneficial in addressing a tumor-promoting inflammatory microenvironment (Figure 1). 

Although the promising antiproliferative potential of UA toward cancer cell lines is well documented, particularly in vitro (Table 1), further research is necessary to reveal the full potential of this compound. Firstly, not all researchers incorporate matched normal cell lines to assess cancer-specific effects. Moreover, the application of a panel of cancer cell lines from different tumor types, such as the NCI-60 Human Tumor Cell Lines Screen panel or cell lines from the Cancer Cell Line Encyclopedia (CCLE) should allow testing UA across diverse cancer types in a high-throughput way or for initial efficacy screening and biomarker discovery. Secondly, the efficacy of UA in vivo, or at least in conditions resembling complex in vivo environments is limited, thus more relevant models should be used. The simplest way to investigate cancer–noncancerous interaction simulating in vivo conditions is a coculture system where the response of cancer cells to a drug is recorded in the presence of additional components of ECM, for instance, fibroblasts or macrophages. Additionally, 3D spheroid cultures are more physiologically relevant models than 2D cultures, and organoids either patient-derived or based on organoid cell lines better recapitulate tumor heterogeneity and microenvironment. Finally, exploration of UA action should be based on animal models, including cell line-derived xenografts (CDX) in which well-characterized cancer cells are implanted in immunodeficient mice or patient-derived xenografts (PDX) where patient tumor fragments are implanted into immunodeficient mice. It is also possible to use genetically engineered mouse models that spontaneously develop tumors, particularly for studies on the chemopreventive activity of UA or its derivatives. 

Overall, the multifaceted actions of UA underscore its potential as a promising therapeutic agent in the fight against various types of cancer, offering a range of mechanisms to disrupt cancer hallmarks and inhibit tumor progression. Further research is needed, not only on the mechanistic aspects of activity and toxicity of UA or UA derivatives and formulations but most of all on biopharmaceutical properties, efficacy and safety in vivo to reconcile the promising great potential of UA with the current lack of its therapeutic use in cancer patients.

## Figures and Tables

**Table 1:** Mechanisms of action of UA toward cancer cells of different origin in vitro and in vivo. UA used in the research was purified from lichens (source is indicated) or synthetized (commercially available, UA).

Organ/Tissue	Cell Lines	Compound Concentrations Tested/IC_50_	Effects In Vitro	Effects In Vivo	References
Head	human glioblastoma cell lines: A172 T98G	(−)-UA extracted from *Cladonia uncialis* IC_50_ = 91.4 ± 2.0 μM IC_50_ = 37.8 ± 3.8 μM (48 h, MTT)	UA dose-dependently decreased the viability of cancer cells. It inhibited the activities of IDO1, COX2, hyaluronidase, SOD, GR and GPx in in vitro assays. Results of the Parallel Artificial Membrane Permeability Assay indicated that UA can cross the blood–brain barrier.		[51]
	human glioblastoma cells U87MG primary rat cerebral cortex cells PRCC	UA IC_50_ = 41.55 µg/mL IC_50_ =132.69 μg/mL (48 h, MTT)	UA in a dose-dependent manner lowered viability and increased LDH release, especially in cancer cells. It revealed high antioxidant capacity in healthy cells (max at 10 µg/mL). UA non-significantly increased 8-OH-2′-deoxyguanosine levels in cancer cells.		[54]
	human oral carcinoma cells KB Normal fibroblasts HGF-1	UA IC_50_ = 30 μM (24 h, MTT)	UA in a dose-dependent manner reduced the viability of KB cancer cells, and normal fibroblasts (HGF-1) were significantly more resistant. UA at concentrations of 10, 20 or 30 μM elevated ROS level, lipid peroxidation, decreased SOD, CAT, GPx activities and reduced GSH level and MMP. It induced DNA damage, apoptosis with the downregulation of Bcl-2 and upregulation of p53, Bax, caspases 9 and 3. It decreased NF-κB, TNF-α and IL-6 levels.		[58]
Lung	non-small cell lung cancer cells A549	UA 25, 50, 100 μM (24 and 48 h trypan blue)	UA decreased viability in a dose- and time-dependent manner, induced G_0_/G_1_ cell cycle arrest with a drop in CDK4, CDK6, cyclin D1 and an increase in p21 levels, mitochondrial membrane depolarization (at 100 μM) and induced apoptosis.		[35]
	non-small cell lung cancer cells A549 (and a panel of other cancer and noncancerous cells)	UA isolated from *F. cucullata* 12.5, 25, 50, 100 μM	UA induced S or G_0_/G_1_ arrest dependent on concentration, apoptosis, decreased Bcl-xL:Bax ratio, reduced clonogenic potential (10 μM) and anchorage-independent growth, motility (5 and 10 μM) and invasion (10 μM). UA elevated E-cadherin (at mRNA ad protein level), reduced mRNA for N-cadherin, Twist and Snail (10 μM); reduced p-c-jun, p-Akt and p-ERK1/2.	Tumor-free survival of BALB/c nude mice with subcutaneously injected A549 was longer if cells were pretreated with the sublethal concentration of UA (10 μM).	[31]
	non-small cell lung cancer cells: A549 H460 H1650 H1975	(+)-UA IC_50_ = 65.3 ± 0.65 μM n/a n/a n/a	5 μM UA reduced transcriptional activity of β-catenin/LEF and AP-1; reduced mRNA for CD44, cyclin D, c-myc; decreased GTP-Rac1 and GTP-RhoA levels, inhibited motility and invasion of lung cancer cells; potentiated activity of cetuximab in inhibiting invasive potential.		[25]
	lung squamous carcinoma cells H520 Calu-1	(+)-UA IC_50_ = 32.51 ± 0.44 μM IC_50_ = 34.25 ± 0.05 μM (48 h, MTT)	UA induced dose-dependent ROS (10, 20 40 μM) and ROS-dependent apoptosis, inhibited mitochondria respiratory chain complexes I and III, decreased Nrf2 protein level and its transcriptional activity (drop in expression of its target genes, HO1 and NQO1), which was mediated by PI3K/Akt pathway inhibition. UA at 15 μM enhanced the cytotoxic activity of paclitaxel (at 0.1 μM) in vitro.	Tumor growth in athymic nude mice inoculated with H520 cells was significantly reduced by UA (50 mg/kg, thrice weekly i.p.) compared with controls. UA at such a dose enhanced the effect of paclitaxel (10 mg/kg, thrice weekly i.p.)	[50]
Breast	human breast cancer cell lines MCF-7 MDA MB 231	UA IC_50_ = 18.9 μM IC_50_ = 22.3 μM	The antiproliferative activity of UA did not involve DNA damage or p53 activation. Although there was an accumulation of p53 and p21 proteins in UA-treated MCF-7 cells, the transcriptional activity of p53 remained unaffected, and there was no phosphorylation of p53 at Ser15.		[53]
	human breast cancer cells T47D	(+)-UA isolated from *Cladonia arbuscula* IC_50_ = 4.2 µg/mL (−)-UA from *Alectoria ochroleuca* IC_50_ = 4.0 µg/mL (24 h, [3H] thymidine incorporation)	Both enantiomers were equally effective in inhibiting cell proliferation. (+)-UA induced G_0_/G_1_ cell cycle arrest and decreased MMP. There was no evidence of apoptosis of cells treated with 20 µg/mL after 24 h or necrosis of cells treated with 5 and 10 µg/mL UA for 24 or 48 h.		[43]
	human breast cancer cells T47D	(+)-UA isolated from *Cladonia arbuscula* 5 and 10 µg/mL tested	UA decreased ATP level after the 24-h exposition. It resulted in activating phosphorylation of AMPK, decreased mTOR/S6K signaling, upregulation of p-eIF2α and induction of autophagy. Autophagy flux was impaired due to the disruption of lysosomal acidification.		[75]
	human medullary breast cancer cells Bcap-37 human umbilical vascular endothelial cells HUVEC	UA 1–50 μM (48–72 h, MTS)	UA dose-dependently inhibited the proliferation of Bcap-37 cancer cells and HUVEC endothelial cells. At 1, 10, 20 μM concentrations, UA inhibited migration and capillary structure formation by HUVEC cells, induced their apoptosis and inhibited activation of VEGFR2 and Akt/p70 S6K/S6 and MEK/ERK1/2 signaling pathways.	In nude mice xenografted with Bcap-37 cancer cells and treated intralesionally with 60 mg/kg/day (22 days) of UA, tumor growth and angiogenesis were inhibited.	[60]
	human breast cancer cells MCF-7 MDA MB 231 SKBR-3 normal human mammary epithelial cells MCF-10A	UA IC_50_ = 34.12 ± 1.25 μM IC_50_ = 38.41 ± 1.64 μM IC_50_ = 48.07 ± 1.52 μM (24 h, MTT)	UA decreased the viability of cancer cells in a dose- and time-dependent manner, while up to 25 μM did not affect normal MCF-10A cells. UA induced apoptosis in MCF-7 cells through the mitochondrial pathway, increased the Bax:Bcl-2 ratio, reduced MMP, increased ROS (25 μM for 24 h) and activated JNK.	UA dose-dependently suppressed tumor growth in nude mice xenografted with MCF-7 cells (i.p. at 25, 50 or 100 mg/kg every 2 days for 21 days). The highest dose (100 mg/kg) was more effective for cancer cells and much less toxic than cyclophosphamide CTX (25 mg/kg).	[49]
	human breast cancer cell lines MCF-7 T47D MDA MB 231 MDA MB 468 SKBR-3 BT-474	UA IC_50_ = 11.2 μM IC_50_ = 15.9 μM IC_50_ = 13.1 μM IC_50_ = 13.7 μM IC_50_ = 14.4 μM IC_50_ = 15.1 μM (72 h, MTT)	UA at 15 and 25 μM induced autophagy in MCF-7 and MDA MB 231 cells, which correlated with a drop in p-Akt, p-4E-PB1 and p-S6K. It inhibited migration and invasion (5–30 μM) of MDA MB 231 cells. Its benzylidene derivative 52 was much more potent in vitro and in vivo.	UA benzylidene derivative 52 was tested in vivo. It inhibited MDA MB 231 and MCF-7 cells xenografted to nude mice (10 mg/kg bw, i.p. 3 times per week).	[32]
	human breast cancer cell lines MCF-7 MDA MB 231 BT-474	UA IC_50_ = 13.11 ± 0.01 μM IC_50_ = 12.84 ± 0.01 μM IC_50_ = 12.65 ± 1.00 μM (48 h, MTT)	Differentially expressed UA-responsive miRNAs were identified and they appeared almost unique to each cell line. The targets are enriched in basal cell carcinoma, MAPK and Hedgehog signaling pathways (MCF-7 cells), ErbB and mTOR signaling, focal adhesion and gap junctions (in BT474 cells), MAPK, ErbB2, PI3K-Akt and p53 signaling pathways (MDA MB 231). Among the upregulated miRNA was a tumor suppressor: has-miR-185-5p.		[46]
	human breast cancer cells SK-BR-3 normal breast epithelial cells MCF-12A	UA IC_50_ = 7.21 μM (48 h, MTT)	UA dose-dependently decreased cancer cell viability with no effect on normal cells. It modulated the expression of apoptosis-related genes, such as these coding for caspases, BCL-, TRAF- and TNF-family members and increased Bax, caspase 3 and 9 protein levels when applied at 7.21 μM for 48 h.		[45]
	human breast cancer cells MCF-7	UA LD_50_ = 13.11 μM	UA decreased NO, VEGF, PGE2 levels, gene expression levels of COX-2 and iNOS and cytokines (IL 2, CXCL10, CXCL8, CCL2, TNF-α, IL-6). It decreased glutathione levels and increased MDA levels in a dose-dependent manner.		[68]
	mouse mammary cancer cells 4T1	UA, HA-UA-GNPs and UA-GNPs IC_50_ = 120.04 ± 4.8 μM IC_50_ = 0.56 ± 2.8 μM IC_50_ = 92.64 ± 3.6 μM, respectively (24 h, MTT)	The cytotoxicity and cellular uptake of UA loaded into gliadin nanoparticles (GNPs) functionalized with hyaluronic acid (HA, targeting CD44 receptor) was higher than UA or UA-GNPs.	Tumor (4T1 cells) growth in BALB/C female mice was efficiently reduced by HA-UA-GNPs, compared with an equal dose (100 mg/kg of UA as an i.p. injection every two days for 21 days) of non-targeted UA-GNPs and free UA.	[76]
Liver	human hepatocellular carcinoma cells HepG-2	UA 1.56–50 μM	UA in time- and dose-dependent manner decreased cell viability, at 24 and 48 h it induced LDH release, and after 24 h induced S phase arrest and apoptosis. It decreased antiapoptotic proteins (Bcl-2, Mcl-1) and reduced activating phosphorylation of Akt, PDK1, mTOR and its substrates (S6K and 4E-BP1). UA elevated autophagy (induction and flux), which was a protective mechanism. UA elevated phosphorylation of ERK1/2, p38 and JNK. The latter kinase was involved in autophagy and apoptosis regulation.		[29]
	human hepatocellular carcinoma cells HepG-2 (HBV(-)) SNU-449 (HBV(+)) human umbilical vascular endothelial cells HUVEC	UA 6.25,12.5, 25, 50, 75 and 100 μM	UA at lower concentrations, 6.25 and 12.5 μM for HepG2 and 6.25 μM for SNU-449, increased viability measured after 24 h. Longer treatment (48 h) was connected with dose-dependent viability drop. UA induced G_0_/G_1_ cell cycle arrest in HepG2, S and G_2_/M arrest in SNU-449, apoptosis and autophagy in both cancer cell lines with limited effect on normal control cells (HUVEC).		[30]
Stomach	human gastric carcinoma cells BGC823 SGC7901	(+)-UA IC_50_ = 236.55 ± 11.12 μM IC_50_ = 618.82 ± 1.77 μM (24 h, CCK-8)	UA induced G_0_/G_1_ cell cycle arrest in BCG823 (100, 200, 400 μM, 24 h) and G_2_/M arrest in SGC7901 (300, 600, 1200 μM), apoptosis with the rise in Bax, cleaved PARP and caspase 3 and a decrease in Bcl-2 levels. UA induced autophagy (elevated LC3-II and decreased p62 levels).	BGC823-bearing nude mice were treated with 100 mg/kg UA i.p. for 11 days (every 2 days), tumor volume and mass were 2-fold lower than control, PBS-treated mice. UA was more effective than 5-FU (25 mg/kg).	[33]
	human gastric adenocarcinoma cells AGS SNU-1	UA 10–50 μM	UA in a dose- and time-dependent manner decreased cell viability, clonogenicity and elevated apoptosis. It reduced MMP and increased Bax:Bcl-2 ratio. In AGS cells UA increased ROS generation in a time-dependent manner and DNA damage was detected by alkaline comet assay after 48 h treatment. UA (15 or 25 μM) in ROS-dependent manner upregulated p-ATM, γ-H2A.X, DNA-PKcs, p53, Chk-2 levels. NAC protected against these effects.		[55]
Pancreas	human pancreatic adenocarcinoma cells Capan-2	(+)-UA isolated from *Cladonia arbuscula* IC_50_ = 5.3 µg/mL (−)-UA from *Alectoria ochroleuca* IC_50_ = 5.0 µg/mL (24 h, [3H] thymidine incorporation)	Both enantiomers were equally effective in inhibiting cell proliferation. (+)-UA induced G_0_/G_1_ cell cycle arrest, decreased MMP. No evidence of apoptosis of cells treated with 20 µg/mL after 24 h, and necrosis was detected in cells treated with 5 or 10 µg/mL UA for 48 h.		[43]
Colon	human colon adenocarcinoma cells HT-29	(+) UA, IC_50_ = 99.7 ± 18.8 μM (72 h, MTT)	UA at 50 or 100 μM in a time-dependent (24, 48 and 72 h) manner decreased MMP and induced apoptosis. It was preceded by ROS elevation (observed after 1, 3 or 6 h post-treatment).		[27]
	human colorectal cancer cell lines HCT116 LS174	(+)-UA 2, 4, 8 μM tested	8 μM UA for 24 or 48 h inhibited SCF-induced cell proliferation and migration; decreased cellular ATP content and increased LDH release. It inhibited mTOR signaling (drop in p-S6K, p-4E-BP) and PKC-A. It elevated autophagy (LC3-II), which was responsible for UA-induced reduction in c-KIT receptor.		[63]
	human colorectal cancer cell lines HCT116 DLD1 SW480 HT29 SW620 Caco2 COLO320 Mouse colorectal cancer cells CT26	UA and potassium usnate (KU), a water-soluble usnic acid salt; 12.5–100 μM tested	UA reduced the viability of cells in a dose-dependent manner; at a 5 μM concentration, it reduced invasion in vitro. KU was more effective in the majority of tested cells. Both UA and KU decreased mRNA for N-cadherin, Snail, Twist, Slug and ZEB2 and protein levels of Twist, Snail and Slug EMT markers in Caco2 cells. KU decreased expression of genes related to motility (*CAPN1*, *CDC42*, *CFL1*, *IGF1*, *WASF1*, *WASL*) in Caco2 cells (UA only affected *CFL1* and *IGF1*).	Firefly luciferase-expressing CT26 cells were inoculated via splenic injection to form multiple tumor foci in the livers of male BALB/c mice. UA at 5 or 10 mg/mL i.p. (6 or 10 times for 2 weeks) or KU at a dose of 5, 10 or 20 mg/kg/mouse i.p. (6 times for 2 weeks) were applied. KU exhibited more potent anticancer effects (PARP cleavage, caspase 3 activation, reduction in EMT markers) and at 20 mg/kg inhibited liver metastasis in an orthotopic murine colorectal cancer model.	[62]
	human colorectal cancer cell lines Caco2, HCTT116 HT29 human gastric cancer cells AGS, MNK45, SNU638	KU-potassium usnate IC_50_ = 38.9 ± 1.76 μM IC_50_ = 56.5 ± 1.49 μM IC_50_ = 103.5 ± 0.76 μM IC_50_ = 41.3 ± 1.61 μM IC_50_ = 120.8 ± 0.51 μM IC_50_ = 46.4 ± 1,63 μM (24 h, MTT)	The viability of a panel of cell lines was dose- and time-dependently reduced by KU. The 24 h treatment with KU at IC50 concentrations induced cell cycle arrest in the G_0_/G_1_ or S phase, depending on the cell line, reduced CDK4, cyclin D2 and transiently elevated p21 protein levels. It induced apoptosis by the mitochondrial pathway. In SNU638 and HCT116 cells KU induced ER stress with the elevation of intracellular Ca^2+^, ROS and ER stress markers, such as BIP, PERK, IRE1α, p-eIF2a, CHOP and ATF3 proteins as well as ATF3-regulated genes. Downregulation of ATF3 by specific siRNA protected against KU-induced elevation of Bak, p-BAD, PUMA, activation of caspase 3 and cell death.	KU applied (20 mg/kg i.p. injections for 16 days) to mice with CT26 metastatic colon cancer cells reduced the number of metastatic nodules in livers, elevated ATF3 and cancer cell apoptosis levels.	[38]
	human colon carcinoma cells RKO	UA 0.5, 1, 5, 10 μM tested	UA at 5 or 10 μM potentiated the inhibitory effect of H_2_O_2_ (400 mM) on the proliferation and migration of RKO cells. Combined treatment enhanced DNA damage, ATM, p-ATM and γ-H2AX elevation, G_2_/M cell cycle arrest, apoptosis and autophagy and elevated ROS. ATM level was controlled by UA-upregulated mir18a-5p.		[56]
	human colorectal cancer cells Caco2 HCT116	UA 2.5, 5, 10, 20 μM tested	UA time- and dose-dependently reduced 14-3-3 proteins, which depended on proteasome and autophagy. It correlated with decreased p-cdc2 level and G_0_/G_1_ arrest. UA at 5 μM decreased invasion in cells expressing different isoforms of 14-3-3 as well as EMT markers, Snail, Twist, N-cadherin, β-catenin (at 10 μM). Among other downregulated proteins were cyclin D1, cyclin B1, p-mTOR, p-Akt, p-STAT3 and p-JNK. UA also reduced the activity of AP-1, STAT and NF-kB transcription factors, which were elevated by overexpression on 14-3-3 isoforms in HEK293T cells. UA inhibited glycolysis and mitochondrial respiration in CaCo2 and HCT116, which correlated with decreased expression of metabolic genes and drop in SLC2A1, HK2, PKM2 and LDHA proteins, even when these processes were elevated by the surplus of an isoform of 14-3-3.		[37]
Prostate	human prostate cancer cell lines PC-3 DU-145 normal prostate epithelial cells PNT2 Skin fibroblasts HSF	UA isolated from *Cladonia arbuscula* (Wallr.) EC_50_ = 2.67 μg/mL EC_50_ = 8.6 μg/mL EC_50_ = 18.2 μg/mL EC_50_ = 20.5 μg/mL (48 h, cell number)	UA inhibited the proliferation of both prostate cancer cells and induced apoptosis of PC-3 cells (cleavage PARP, caspase 7 and 9 elevations). UA induced actin cytoskeleton rearrangements in a dose-dependent manner in both cancer cell lines and reduced DU-145 cell motility.		[40]
	human prostate cancer cells DU-145	UA IC_50_ = 42.15 ± 3.76 μM (48 h, MTT)	UA decreased cell viability in a dose- and time-dependent manner, reduced MMP, elevated Bax:Bcl-2 mRNA, activated apoptosis and downregulated NF-κB p50.		[41]
Ovaries	human ovarian adenocarcinoma cells A2780	(+)-UA IC_50_ = 75.9 ± 2 μM (72 h, MTT)	At 50 or 100 μM in a time-dependently (48 and 72 h) increased S phase cell cycle arrest, UA decreased MMP and induced apoptosis. It was preceded by RNS elevation (observed after 3 or 6 h post-treatment).		[26,27]
	human ovarian adenocarcinoma cells lines OVCAR-3 A2780 mouse fibroblasts L929	(+)-UA IC_50_ = 20 μM xCELLingence system and MTT	UA in a dose- and time-dependent manner reduced the viability of cancer cells while normal fibroblasts were more resistant (24 h). 20 μM UA induced G_0_/G_1_ cell cycle arrest and apoptosis, inhibited migration and invasion of OVCAR-3 cells. UA modulated transcriptome, particularly elevated expression of some genes connected with apoptosis (caspase 1 and 8, for instance).		[44]
Uterus	human cervical cancer cell lines HeLa SiHa CaSKi (and other types of cancer cells) normal human cervical epithelial cells HcerEpic human umbilical vascular endothelial cells HUVEC	UA 3, 10, 30 and 100 μM tested (24 h, MTT)	UA dose-dependently reduced PD-L1 levels in a panel of cancer cells, including HeLa. It inhibited PD-L1 protein synthesis and enhanced its degradation in HeLa cells, which correlated with its lower level at the cell surface and enhanced T-lymphocyte killing activity toward cervical cancer cells. It inhibited mTOR, which induced autophagy and autophagic degradation of PD-L1. UA inhibited Jak1/2-Src-STAT3 and Ras-MEK-ERK pathways leading to reduced PD-L1 expression, a drop in c-myc and cyclin D1 levels and reduced clonogenic potential. UA diminished the PD-L1-mediated angiogenic potential of HUVEC cells.		[36]
	human cervical cancer cells HeLa	UA isolated from *Usnea cornuta* IC_50_ = 48.65 μM (24 h, MTT)	UA at 25 and 50 μM increased ROS levels, lipid peroxidation, decreased MMP and GSH levels and increased caspase3/7 activity and cell death. UA induced protective autophagy—its inhibition by chloroquine increased UA cytotoxicity.		[52]
	endometrial cancer cells Ishikawa	UA IC_50_ = 51.76 μM (48 h, XTT)	UA inhibited cell proliferation and downregulated the expression of oncogenic lncRNA UCA1.		[48]
Blood	human acute myeloid leukemia cells HL-60 human chronic myelogenous leukemia cells K562	UA IC_50_ = 10.00 ± 1.03 μM IC_50_ = 10.39 ± 0.60 μM (3 days, cell number)	UA induced apoptosis in human leukemia cells with HL-60 cells being more responsive. It correlated with caspase 3, 9 and 8 activation, PARP cleavage and drop in Mcl-1. UA inhibited Mnk1/eIF4E and Pim1/4E-BP1 signaling, increased p27 and decreased cyclin D1, p-Bad, c-myc, Pim-1 levels. UA inhibited Pim-1 activity in vitro.		[39]
Skin	human melanoma cells HBT-140 Skin fibroblasts HSF	UA isolated from *Cladonia* *arbuscula* (Wallr.) EC_50_ = 13.7 μg/mL EC_50_ = 19.3 μg/mL (72 h, cell number)	UA exerted weak cytostatic effects and apoptosis induction. At 10 and 25 μg/mL, it induced rearrangements of the actin cytoskeleton in a dose-dependent manner, 10 µg/mL UA inhibited cell motility.		[40]
	human melanoma cell lines HTB140 A375 WM793 Murine macrophages RAW264.7	(+)-UA and (−)-UA IC_50_ = 14.7 and 20.6 µg/mL IC_50_ = 11.8 and 22.2 µg/mL IC_50_ = 30.1 and 52.1 µg/mL respectively (48 h, LDH)	Both enantiomers decreased the viability and proliferation of cells in a dose- and time-dependent manner, but their potency was enantiomers and cell-line specific. They inhibited cell migration (at 10 µg/mL) and acted synergistically with doxorubicin in A375 cells. They weakly decreased the release of pro-inflammatory TNF-a, IL-6 and NO and significantly reduced the synthesis of TLR4, cPLA2, COX-1 and COX-2 in LPS-stimulated macrophages (10 or 25 µg/mL concentrations tested).		[20]
	squamous cell carcinoma A-431 normal human embryonic kidney cells HEK293T	UA IC50 = 98.9 ± 6 μM (48 h, MTT)	UA induced dose-dependent cytotoxicity (within the range 25–250 μM) in cancer but not in normal cells. It induced LDH release and PI accumulation by cancer cells. It correlated with ROS elevation, lipid peroxidation, changes in surface lipids and proteins, drop in GSH level and MMP. UA induced G_0_/G_1_ cell cycle arrest and apoptosis.		[34]
